# Supplementary material for: Health inequity associated with financial hardship among patients with kidney failure
Source: PLoS One. 2023 Jun 23;18(6):e0287510. doi: 10.1371/journal.pone.0287510 (PMC10289308; doi:10.1371/journal.pone.0287510)
Supplement: S1 File — This form was developed by the research team to collect demographic information of the participants. (PDF) [file pone.0287510.s001.pdf]

Supporting Information 1

背景資料問卷 DEMOGRAPHIC FORM

1. 性別 Sex： ☐ 男 Male ☐ 女 Female
2. 年齡 Age： ______
3. 腎病原因 Cause of kidney failure： ☐ 糖尿病 Diabetes ☐ 高血壓 Hypertension ☐ 腎炎 Nephritis
   ☐ 其他泌尿系統疾病 Other urological disorder ☐ 其他 Others：______
4. 每日更換透析液次數 Daily frequency of fluid change： ______ 或 or ☐ 使用自動腹膜透析機 Using APD
5. 開始接受透析日期 Dialysis start day： ______年 Year______月 Month
6. 曾否接受腎臟移植 History of kidney transplantation： ☐ 沒有 No ☐ 有 Yes
7. 婚姻狀況 Marital status： ☐ 單身 Single ☐ 已婚 Married ☐ 離婚 Divorced ☐ 其他 Others：______
8. 就業情況 Employment status： ☐ 全職 Full-time ☐ 兼職 Part-time ☐ 失業 Unemployed ☐ 其他 Others：______
9. 職業 Occupation： ____________
10. 最高教育程度 Highest education level： ☐ 未曾接受正式教育 No formal education ☐ 小學以下 Below primary ☐ 小學 Primary
    ☐ 中學 Secondary ☐ 專上教育 Tertiary ☐ 學士 Bachelor ☐ 碩士或以上 Master or above
11. 家庭每月收入 Household monthly income： ☐ 少於 Less than $10,000 ☐ $10,000-19,999 ☐ $20,000-29,999
    ☐ $30,000-39,999 ☐ $40,000-49,999 ☐ $50,000 或以上 or above
12. 你有沒有被醫生診斷患上以下長期病而需接受治療或跟進？Have you been diagnosed with the following conditions?
    ☐ 癌症 Cancer ☐ 心臟病 Heart disease ☐ 周邊血管疾病（包括：中風、靜脈栓塞）Peripheral vascular disease ☐ 心臟衰竭 Heart failure
    ☐ 糖尿病 Diabetes ☐ 結締組織疾病（包括：紅斑狼瘡、類風濕關節炎、多發性硬化症）Rheumatological disease
    ☐ 其他嚴重疾病 Other advanced conditions：____________
