# Supplementary material for: Health inequity associated with financial hardship among patients with kidney failure
Source: PLoS One. 2023 Jun 23;18(6):e0287510. doi: 10.1371/journal.pone.0287510 (PMC10289308; doi:10.1371/journal.pone.0287510)
Supplement: Attachment — Submitted filename: PLOSO_Response_221220.docx [file pone.0287510.s002.docx]

We are pleased to re-submit the revised manuscript entitled ‘Health Inequity Associated with Financial Hardship Among Patients with Kidney Failure: A Secondary Analysis’ (Manuscript No.: PONE-D-22-21227). We would like to thank the Editor and Reviewer for their thoughtful comments on the manuscript. Our responses to each of the comments are provided below in italics. The suggested revisions have strengthened the report of preliminary evidence, which informs directions for future research in health equity and financial wellbeing of patients with kidney failure. Thank you for your attention and I look forward to hearing from you.

Title/Abstract
• Line 5/20: Recommend using “kidney failure” rather than “end-stage kidney disease” per guidelines advanced by Levey et al. (2020)
Levey AS, Eckardt KU, Dorman NM, Christiansen SL, Cheung M, Jadoul M, et al. Nomenclature for kidney function and disease: executive summary and glossary from a Kidney Disease: Improving Global Outcomes consensus conference*. Nephrology Dialysis Transplantation. 2020 Jul 1;35(7):1077–84.

*Response: We revised the wordings (e.g., kidney failure, kidney care) accordingly.*

Introduction
• Line 48: The authors state that patients may be required to cover 12-71% of dialysis costs out of pocket, but do not specify the nature of reimbursement at the hospitals in the study. This context would be especially helpful for understanding the impact of financial hardship on study participants. Also, I think it may be relevant to mention here that, as I understand it, public healthcare is virtually free and guaranteed in Hong Kong as this is not the case elsewhere.

*Response: We added a description about Hong Kong’s healthcare system in the Introduction section (p.4 lines 72-79).*

• Lines 53-60: This section seems to emphasize the relationship between financial hardship and medical expenses, but I think the experience of economic oppression is more profound than that. I think the authors can do more to describe the local context of economic inequality in Hong Kong. I am not an expert in the region, though I think some relevant dynamics include issues of financialization, housing and spatial segregation, and immigration issues.

*Response: Thanks for this very thoughtful comment. We added a description about Hong Kong’s economic inequity in the Introduction section (p.4 lines 72-79).*

Materials and Methods
• Line 71: Are you able to add details about the hospitals from which these data were collected? They are described as “regional hospitals” but are they public? Private?

*Response: We provided details about these hospitals in the Materials and Methods section (p.4 lines 82-84).*

• Line 72: Please provide a citation where the inclusion and exclusion criteria are previously reported.

*Response: Details of the original study can be found in the reference #16. We added the inclusion and exclusion criteria in the Methods section for better understanding of the study design (p.4 line 85-87).*

• Lines 93-100: I am not sure that the Methods adequately account for all four instruments and analyses. I also imagine chi-squared analyses were conducted to assess for differences in patient demographics, but these are not reported in the Methods. Looking at Table 1, I see composite scores for the Kidney Disease Quality of Life-36, the Karnofsky Performance Scale, and the Charlson Comorbidity Index, in addition to several individual patient-reported outcomes. Do the patient-reported outcomes derive from the Dialysis Symptoms Index? If so, how and why were only a subsample of the 30 symptoms selected for reporting?

*Response: We supplemented detailed accounts and references for the four instruments used in the Methods section (p.5 lines 92-110). To provide a fair comparison, we presented the results of all symptoms in the Dialysis Symptoms Index and other instruments in Table 1.*

Results
• The authors report significant results but do not describe results that are not significant, which is important.

*Response: Thanks for pointing out this issue. We stated non-significant results in the Results and Discussion sections (p.6 lines 138-139; p.7 lines 148-149) and included the statistics in the Table 1.*

Discussion
• Lines 126-128: I am not sure that this is the most accurate summary statement given that (1) your results differ based on employment status and poverty level, and (2) not all patient-reported outcomes were significantly different between your groups. I would revise this summary statement to provide a more cautious interpretation of your results.

*Response: We revised the summary to precisely capture both significant and non-significant results (p.7 lines 148-152).*

• Lines 132-133: Consider rephrasing to situate the statistics of your study population relative to the general population (e.g., “In this study, roughly half as many people were employed and three times as many lived below the poverty line relative to the general population of Hong Kong”).

*Response: We revised the statements based on your suggestion (p.7 line 154).*

• Lines 137-142: Do you think that people experiencing economic oppression are at higher risk of developing kidney failure or that undergoing hemodialysis impedes employment opportunities, which then leads to financial hardship? Some literature on this might be helpful to include in the introduction. This might also be worth mentioning in the limitations with respect to the need for longitudinal analyses.

*Response: Thanks for your suggestion. We added this point about disparities in the Introduction section (p.3 lines 53-55). We also stressed the need for a longitudinal study to investigate the impact of financial hardship on the outcomes of patients with kidney failure (p.8 lines 181-183).*

• Lines 159-160: Can you recommend additional factors affecting financial hardship (e.g., wealth, homeownership)?

*Response: We made recommendations on additional factors based on our recent review (p.8 lines 180-181).*

Table 1
• The origin of the patient-reported outcomes is not clear to me. Are these from the DSI? If so, why are there not 30?

*Response: We substantially revised Table 1 to present all the results, regardless their statistical significance levels.*

• What is the KDQOL-36 PCS vs. MCS?

*Response: We are sorry about the confusion. We defined all the abbreviations in the legends and provided details of the instruments in the Methods section (p.5 lines 92-110).*

• How did you choose serum albumin vs. urine microalbumin as a clinical outcome?

*Response: Serum albumin was chosen as an indicator of nutritional status but not disease progression. The data were retrieved from the laboratory results in the medical records. We analysed glomerular filtration rate to evaluate disease progression but no significant result was found (Table 1).*
