# Supplementary material for: Health inequity associated with financial hardship among patients with kidney failure
Source: PLoS One. 2023 Jun 23;18(6):e0287510. doi: 10.1371/journal.pone.0287510 (PMC10289308; doi:10.1371/journal.pone.0287510)
Supplement: Attachment — Submitted filename: PLOSO_Response_230224.docx [file pone.0287510.s003.docx]

We are pleased to re-submit the revised manuscript entitled ‘Health Inequity Associated with Financial Hardship Among Patients with Kidney Failure: A Secondary Analysis’ (Manuscript No.: PONE-D-22-21227). We would like to thank the Editor and Reviewer for their thoughtful comments on the manuscript. Our responses to each of the comments are provided below in italics. The suggested revisions have strengthened the report of preliminary evidence, which informs directions for future research in health equity and financial wellbeing of patients with kidney failure. Thank you for your attention and I look forward to hearing from you.

1). Authors have used ttest to compare outcomes between two financial level groups. ttest does not account for confounders, therefore factors that authors identified should be interpreted only as markers not as those with independent effects. Therefore conclusions need to be cautious to be within that limitation.

*Response: Thanks for this kindly advice. The limitation related to the statistical methods was highlighted in the Discussion section.*

2). Line 59/60. “Negative health outcomes, including depression, anxiety, lower health-related quality of life (HRQoL), and higher mortality risk, have been reported (9-11)”. Is this statement referring to those with lower financial levels or those with CKD in general?

*Response: The statement was revised to enhance clarity.*

3). Line 81. How were patients selected? All the patients who met inclusion/exclusion criteria in these 2 hospitals during the recruitment period were included or was there any selection? How representative the participants to non-participants if there was a selection? Was there any reason (like sample size estimation) for selecting specifically N=354 patients?

*Response: Further details about recruitment were provided in the Methods and Results sections.*

4). Line 87-88. “… research assistant administered a questionnaire…”. Please indicate what information were collected through this questionnaire. I assume this questionnaire was used to collect all patient-reported outcomes as opposed to assessing inclusion/exclusion criteria.

*Response: The statement was revised to enhance clarity.*

5). Line 82. “… patients were recruited from two public hospitals”. Could you name these hospitals?

*Response: The names of the hospitals were provided.*

6). Line 89/90. “… approved by the institutional research boards of the university and the involved hospitals”. Could you name these institutions?

*Response: This statement which is redundant was removed. The names of the IRBs involved were provided together with the reference numbers in the following statement.*

7). Line 108-112. Please give references for weighted score version of Charlson Comorbidity Index and MDRD equation. Cited ref 23 not applicable for these measures.

*Response: References were updated for the captioned instruments.*

8). Line 124. 58.5% were male. Different percentage in table1.

*Response: Thanks for spotting out the inconsistency. The figure was checked against raw data and the numbers in Table 1 were revised.*

9). Line 131-141. This paragraph included results of comparing individual symptoms used to derive DSI score. This is a concern for multiple reasons. First, DSI index has been validated as a summative measure, not for individual symptoms, as reported in lines 92-110. Therefore how correct this comparison of individual items? Why validated summative DSI score not compared between groups? Second, ttest need outcome measures to be in continuous scale, patient-reported responses for these individual symptoms are in categorical scales (ie, likert scale coded to numeric) rather than in continuous scale, therefore not suitable for ttest. If authors need to retain these individual symptoms comparisons as results they need a justification with supporting reference(s) on why ttest is suitable. As at present ttest is not an appropriate statistical method. Otherwise comparison has to be done using a statistical method appropriate for the data. Same comment applicable to the corresponding results in table1.

*Response: Thanks for raising the statistical concerns. First, we added the comparison of DSI total scores in Table 1 for reference. The DSI not only generates a score to indicate the overall symptom burden, but it also provides a framework for assessing CKD-related symptoms, as demonstrated by good content validity and test-retest reliability of individual items in the original instrument (Weisbord et al., 2004). Of note, in a study by Weisbord et al. (2007), symptoms reported by patients and clinicians were compared based on the mean scores of individual symptoms. Second, it is implicitly assumed that the individual item scores of DSI are of interval scale data, otherwise it does not make sense to calculate a summative score for DSI. Indeed, it is common to report means and standard deviations of individual symptoms in previous studies (Almutary et al., 2016; de Rooji et al., 2022; Weisbord et al., 2007) and the item scores are not skewed. Therefore, we believe that t-test is appropriate to compare the severity levels of symptoms between groups.*

*References:*

*Almutary, H., Bonner, A., & Douglas, C. (2016). Which patients with chronic kidney disease have the greatest symptom burden? A comparative study of advanced CKD stage and dialysis modality. Journal of Renal Care, 42(2), 73-82.*

*de Rooij, E. N., Meuleman, Y., de Fijter, J. W., Jager, K. J., Chesnaye, N. C., Evans, M., ... & Hoogeveen, E. K. (2022). Symptom burden before and after dialysis initiation in older patients. Clinical Journal of the American Society of Nephrology, 17(12), 1719-1729.*

*Weisbord, S. D., Fried, L. F., Arnold, R. M., Rotondi, A. J., Fine, M. J., Levenson, D. J., & Switzer, G. E. (2004). Development of a symptom assessment instrument for chronic hemodialysis patients: The Dialysis Symptom Index. Journal of Pain and Symptom management, 27(3), 226-240.*

*Weisbord, S. D., Fried, L. F., Mor, M. K., Resnick, A. L., Unruh, M. L., Palevsky, P. M., ... & Arnold, R. M. (2007). Renal provider recognition of symptoms in patients on maintenance hemodialysis. Clinical Journal of the American Society of Nephrology, 2(5), 960-967.*

10). Abstract line 32. “... increased distress associated with specific symptoms,…”. Methods section says dialysis symptom index (DSI) is derived from kidney symptoms, and higher DSI indicates higher distress. If so, above statement is obvious by definition, therefore I wonder why it is worth reporting as a result.

*Response: The meaning of the captioned phrase means distress ‘originated from specific symptoms.’ We did not imply a statistical association between distress and specific symptoms. The statement was revised to enhance clarity.*

11). Table1. Please remove all asterisks and associated footnote because they are redundant.

*Response: All asterisks and footnotes were removed accordingly.*

12). Table1. Please check the correctness of the reported SD (0.11) for age for unemployed group.

*Response: The figures were checked against raw data and the number in the Table 1 was revised.*

13). Table1. Please mention Clinical visits and ER attendance are counted over what period. I could not find that in methods section. Please make sure method section includes how each of the reported measure was measured.

*Response: The duration of capturing clinical/ER visits and lengths of hospital stay were added in the Methods section and Table 1.*
