# Supplementary material for: Health inequity associated with financial hardship among patients with kidney failure
Source: PLoS One. 2023 Jun 23;18(6):e0287510. doi: 10.1371/journal.pone.0287510 (PMC10289308; doi:10.1371/journal.pone.0287510)
Supplement: Attachment — Submitted filename: PLOSO_Response_230418.docx [file pone.0287510.s004.docx]

We are pleased to re-submit the revised manuscript entitled ‘Health Inequity Associated with Financial Hardship Among Patients with Kidney Failure: A Secondary Analysis’ (Manuscript No.: PONE-D-22-21227). We would like to thank the Editor and Reviewer for their thoughtful comments on the manuscript. Our responses to each of the comments are provided below in italics. The suggested revisions have strengthened the report of preliminary evidence, which informs directions for future research in health equity and financial wellbeing of patients with kidney failure. Thank you for your attention and I look forward to hearing from you.

1. Line 22: Please specify the terms “kidney failure patients” in this line “This cross-sectional study aimed to identify the differences in patient-reported and clinical outcomes among patients with different financial status”

*Response: The sentence was revised accordingly.*

2. Line 24: Please start with the word in this line “354 patients with end-stage kidney disease were recruited from 25 March to June 2017 at two regional hospitals in Hong Kong”

*Response: The sentence was revised so that it begins with a word.*

3. Line 66: it may be "aid" instead of "aids."

*Response: The term was revised accordingly.*

4. Line 68: Please add this line “In Hong Kong, not enough research is done to examine health consequences of financial status.”

*Response: We inserted a statement based on this suggestion.*

5. Line 73-75: Can the author give more recent data?

*Response: We updated the latest available figures of per capita GDP and Gini coefficient in 2021 and 2019 (World Bank Group, 2023; World Economics, 2019), respectively.*

References

World Bank Group. GDP per capita (current US$) – Hong Kong SAR, China [Internet]. 2023 [Accessed 18 April 2023]. Available: https://data.worldbank.org/indicator/NY.GDP.PCAP.CD?locations=HK.

World Economics. Hong Kong’s Gini coefficient [Internet]. 2019 [Accessed 18 April 2023]. Available from: https://www.worldeconomics.com/Inequality/Gini-Coefficient/Hong%20Kong.aspx#.

6. Line 92: “After explaining the study and obtaining informed consent, the research assistant administered a questionnaire containing a demographic form and the instruments.” Please share that form as a supplementary file.

*Response: We attached the demographic form as a supplementary material in the revised version.*

7. Line 108: it maybe "It’s" instead of "Its"

*Response: We checked with the editor and confirmed that the use of ‘its’ in this case is appropriate.*

8. Line 110-118: Please specify categories of all outcome (categorical) variables and also how they are categorized.

*Response: The outcomes (i.e., functional status, comorbidity level, healthcare service utilization, biochemical parameters) were analysed as continuous variables and their measurements were described in the paragraph. We have now specified all categories of the categorical variables (i.e., sex, marital status, education level, dialysis modality, history of transplantation) in Table 1.*

9. Line 127-128: Please add a few lines about how you measure the P-value for frequency or percentage information. Did the author check the chi-square test for the categorical variables? Please provide the relevant statistics.

*Response: We added detailed description about comparing background characteristics of patients with different financial status. Specifically, categorical variables were compared between the financial status groups by using chi-squared test, while all patient-reported and clinical outcomes were compared by using independent sample t-test.*

10. Line 166: Why the author didn’t conduct any regression model? Any explanation?

*Response: This study analysed data from the original study on symptom experience of patients with kidney failure. Factors potentially influencing financial hardship (e.g., financial aids, home ownership, personal savings, health spendings) have not been accessed in the original study (Ng et al., 2020; 2021). It is therefore immature to conduct adjusted analysis, such as regression analysis. Exploratory univariate analyses were only conducted aiming to provide some insights into whether there are differences in patient-reported and clinical outcomes in terms of financial hardship.*

References

Ng MSN, Miaskowski C, Cooper B, Hui YH, Ho EHS, Mo SKL, et al. Distinct symptom experience among subgroups of patients with ESRD receiving maintenance dialysis. J Pain Symptom Manage 2020;60:70-9.

Ng MSN, Chan DNS, Cheng Q, Miaskowski C, So WKW. Association between financial hardship and symptom burden in patients receiving maintenance dialysis: a systematic review. Int J Environ Res Public Health 2021;18:9541.

11. Line 131: Please provide this information “58.5% were male” either in a graph or in the table.

*Response: This information was presented in Table 1 (Background characteristics – Overall).*

12. Line 173-175: Can the author justify this?

*Response: We added a sentence to clarify the relationship with the support of a relevant study.*

13. Line 179-182: Please clarify “While symptoms and other outcomes are signs of deteriorating health, patients with a poorer symptom status or poorer outcomes may have a higher demand for healthcare services and a lower physical capacity for engaging actively in employment.”

*Response: The sentence was revised to clarify the reciprocal relationship. While symptoms and other outcomes may be the consequences of financial hardship, they may also reflect a lower physical capacity for engaging in economic activities.*
