# Supplementary material for: Health inequity associated with financial hardship among patients with kidney failure
Source: PLoS One. 2023 Jun 23;18(6):e0287510. doi: 10.1371/journal.pone.0287510 (PMC10289308; doi:10.1371/journal.pone.0287510)
Supplement: Attachment — Submitted filename: PLOSO_Response_230530.docx [file pone.0287510.s005.docx]

We are pleased to re-submit the revised manuscript entitled ‘Health Inequity Associated with Financial Hardship Among Patients with Kidney Failure’ (Manuscript No.: PONE-D-22-21227). We would like to thank the Editor and Reviewer for their thoughtful comments on the manuscript. Our responses to each of the comments are provided below in italics. The suggested revisions have strengthened the report of preliminary evidence, which informs directions for future research in health equity and financial wellbeing of patients with kidney failure. Thank you for your attention and I look forward to hearing from you.

1. Although the authors tried to give an explanation, which is not quite satisfactory. I think a logistic or linear regression analysis was possible by dichotomizing the KPS scale scores or taking it as a continuous scale, where important factors age, sex, marital status, employment, education years, dialysis types, history of transplantation, days of hospital stay could have been taken as factor variables, in order to check the nature and magnitude of association of financial hardship and health when adjusted for other factors.

*Response: We used multiple regression analyses to examine the associations between outcome variables, employment status, and income level adjusted for demographic factors (sex, marital status, education level, dialysis modality, history of transplantation, age, duration on dialysis). Findings were presented in Tables 1 and 2. The relevant contents in the Results, Discussion, and Abstract were amended accordingly.*

2. In addition, from the methods it seems clear that some information was collected directly from patients. Hence, the words “A secondary analysis” in the title does not seem appropriate.

*Response: We deleted the term ‘secondary analysis’ from the title and Methods.*

3. The author should explore the said analyses, add additional tables and description and add necessary discussion.

*Response: We conducted regression analyses, and their details and implications were added in the Methods, Results, and Discussions as appropriate.*
