# Supplementary material for: Health inequity associated with financial hardship among patients with kidney failure
Source: PLoS One. 2023 Jun 23;18(6):e0287510. doi: 10.1371/journal.pone.0287510 (PMC10289308; doi:10.1371/journal.pone.0287510)
Supplement: Attachment — Submitted filename: PLOSO_Response_230606.docx [file pone.0287510.s006.docx]

We are pleased to re-submit the revised manuscript entitled ‘Health Inequity Associated with Financial Hardship Among Patients with Kidney Failure’ (Manuscript No.: PONE-D-22-21227). We would like to thank the Editor and Reviewer for their thoughtful comments on the manuscript. Our responses to each of the comments are provided below in italics. The suggested revisions have strengthened the report of preliminary evidence, which informs directions for future research in health equity and financial wellbeing of patients with kidney failure. Thank you for your attention and I look forward to hearing from you.

1. The following variables are not normally distributed in your data: Months on dialysis, CCI, clinical visits in 6 months, ER attendance in 6 months and days of hospital stay in 6 months. It is not justified to use t test for comparison of these variables across income and employment groups. Also, data should be expressed as 'median (IQR)' rather than as mean SD. So, for these variables in the tables do following things.
   a. Use Wicoxon Rank Sum Test (Mann-Whiteny U test) for comparison
   b. Express as Median (IQR) for all three columns
   *Response: We appreciate these precise and constructive suggestions. We conducted the analyses as advised and revised the relevant parts in the manuscript (including Analyses, Results, and Tables).*
2. Instead of writing N % and Mean SD in the table rows, explain how data was presented in the footnotes. For example you can write- Data was presented as N %, Mean SD and Median IQR where appropriate.
   *Response: We added a footnote to indicate variables of which medians and inter-quartile ranges are presented.*
3. Remove the column containing t values from the table. The p-values expressed in three decimal points are enough to display the student distribution of the differences.
   *Response: The column was deleted as advised.*
4. Give the full forms of abbreviations used in the tables in table footnotes. (E.g. PCS, MCS, KPS, CCI etc).
   *Response: We provided the full names of the instruments in the Tables.*
